# Bloodstream Infection and Hospital Outcomes Among Very Low Birth Weight Discharges: Mortality, Mechanical Ventilation, and Prolonged Hospitalization in the 2022 HCUP Kids’ Inpatient Database

**DOI:** 10.3390/children13091182

**Published:** 2026-09-02

**Authors:** Michael Samawi, Hani Samawi, Gulzar H. Shah, Majd Al-Saleh

**Affiliations:** 1 Defense Language Institute Foreign Language Center, 1759 Lewis Road, Monterey, CA 93944, USA; 2 Jiann-Ping Hsu College of Public Health, Georgia Southern University, 501 Forest Drive, Statesboro, GA 30458, USA; hsamawi@georgiasouthern.edu (H.S.); gshah@georgiasouthern.edu (G.H.S.); ma22524@georgiasouthern.edu (M.A.-S.)

**Keywords:** neonatal bloodstream infection, very low birth weight, NQI03, HCUP Kids’ Inpatient Database, mechanical ventilation, mortality, length of stay, selection bias, multiple imputation

## Abstract

**Highlights:**

**What are the main findings?**

- Among 39,630 VLBW discharges, NQI03 numerator-code-positive bloodstream infection was associated most strongly with mechanical ventilation and prolonged hospitalization. Secondary complete-case standardized absolute differences were 15.9 and 5.9 percentage points, respectively, versus 1.0 point for mortality.
- Half of all in-hospital deaths occurred during stays shorter than three days; imposing the NQI03-like short-stay criterion increased the mortality aOR from 1.20 to 1.96.

**What are the implications of the main findings?**

- NQI03 numerator-code-positive bloodstream infection is an administrative discharge-level construct with unknown temporal ordering and should not be interpreted causally.
- Quality indicator eligibility rules such as the three-day LOS criterion can materially alter patient-level outcome associations and should be evaluated before reuse as analytic cohort restrictions.

**Abstract:**

**Background/Objectives**: We examined discharge-level associations between an administrative NQI03 numerator-code-positive bloodstream infection construct and hospital outcomes in very low birth weight (VLBW) discharges and whether the NQI03 short-stay criterion altered mortality estimates. **Methods**: Using the 2022 HCUP Kids’ Inpatient Database, we constructed an independent 500–1499 g cohort from ICD-10-CM birth-weight codes. Mortality, procedure-defined mechanical ventilation, and prolonged hospitalization (≥108 days, cohort P90) were modeled with adjustment for birth weight, completed gestational age, patient/discharge characteristics, and hospital characteristics. Missing baseline covariates were addressed with 50 multiple imputations; survey-design, transfer/inborn, short-stay, and model-specification sensitivities were examined. **Results**: Among 39,630 discharges from 1750 hospitals, 1655 (4.18%) were NQI03 numerator-code-positive. Multiple-imputation aORs were 1.20 (95% CI 1.005–1.432) for mortality, 2.50 (2.14–2.93) for mechanical ventilation, and 2.02 (1.75–2.33) for prolonged hospitalization; corresponding adjusted risk ratios were 1.17, 1.29, and 1.60. Secondary complete-case record-level standardized probabilities were 7.5% versus 6.5%, 58.6% versus 42.6%, and 15.4% versus 9.5%, respectively; uncertainty for these estimates is reported using hospital-cluster bootstrap confidence intervals. Of 3041 deaths, 1528 (50.25%) occurred during stays <3 days; imposing LOS ≥ 3 days increased the mortality aOR to 1.96. **Conclusions**: NQI03 numerator-code-positive bloodstream infection showed a modest, imprecise association with mortality and more pronounced associations with mechanical ventilation and prolonged hospitalization. Mortality estimates were sensitive to short-stay eligibility. Because infection timing is unavailable, findings are non-causal discharge-level associations.

## 1. Introduction

Bloodstream infection remains a major source of morbidity and mortality in neonatal intensive care, particularly among very low birth weight and very preterm newborns [1,2]. Clinical severity tools can characterize physiologic deterioration but require laboratory and bedside measures that are unavailable in large administrative discharge databases [3,4]. Administrative data therefore offer breadth at the cost of temporal and physiologic detail.

The Agency for Healthcare Research and Quality (AHRQ) Neonatal Quality Indicator 03 (NQI03) identifies neonatal bloodstream infection through ICD-10-CM/PCS logic within a specified denominator [5]. Our 2019 KID analysis examined patient and hospital characteristics associated with the occurrence of NBSI [6]. The present study addresses a different question: among VLBW discharges, how is the NQI03 bloodstream infection numerator coding construct associated with adverse hospital outcomes?

This distinction matters because quality indicator eligibility rules are not necessarily neutral sampling rules for patient-level outcome research. The NQI03 denominator excludes stays shorter than three days and contains eligibility pathways involving death, mechanical ventilation, surgery, or transfer [5]. Conditioning an outcome analysis on such rules can alter the composition of the comparison groups. We therefore constructed the VLBW cohort independently of the NQI03 denominator and applied only the published NQI03 numerator diagnosis-code logic as the exposure.

Our primary objective was to estimate the adjusted association between NQI03 numerator-code-positive bloodstream infection and in-hospital mortality. Secondary objectives were to estimate corresponding associations with procedure-defined mechanical ventilation and prolonged hospitalization, to provide adjusted absolute outcome probabilities and risk ratios, and to examine robustness to missing-data handling, KID survey design, transfer/inborn status, gestational age specification, alternative LOS thresholds, and the NQI03 short-stay criterion. Hospital, payer, and race/ethnicity coefficients were treated as exploratory. The study was designed to inform both clinical interpretation of these discharge outcomes and methodological use of neonatal quality indicators in administrative-data research.

## 2. Materials and Methods

### 2.1. Data Source and Study Design

We performed a retrospective cross-sectional analysis of the 2022 HCUP Kids’ Inpatient Database (KID), an all-payer pediatric discharge sample produced by AHRQ [7,8]. The 2022 KID Core contains 3,009,812 discharge records. Hospital characteristics were linked from the KID hospital file by HOSP_KID. The unit of analysis was a discharge rather than a unique infant; repeated admissions cannot be linked reliably in KID.

This study was reviewed by the Georgia Southern University Institutional Review Board and determined to be exempt (protocol H26358; approved 24 June 2026). KID data are de-identified. Analyses were performed in SAS 9.4 (SAS Institute Inc., Cary, NC, USA). Reporting followed the STROBE framework and the RECORD extension for studies using routinely collected health data [9].

### 2.2. Independent VLBW Cohort

The primary cohort was constructed directly from raw ICD-10-CM diagnosis fields rather than from the NQI03 denominator. All 40 diagnosis positions (I10_DX1-I10_DX40) were searched for AHRQ Appendix-L birth-weight codes corresponding to 500–749, 750–999, 1000–1249, and 1250–1499 g [5]. If more than one birth-weight category was present, the lowest category was retained. No LOS, survival, ventilation, surgery, transfer, neonatal-age, or NQI03 denominator restriction was used to enter the primary cohort.

This design was selected because the NQI03 denominator excludes stays shorter than three days and therefore removes a substantial fraction of early deaths. An exact reconstruction of every NQI03 newborn/outborn denominator element was not used as the primary cohort because AGEDAY, ATYPE, and POINTOFORIGINUB04 are not available in the KID Core in the form required by the indicator specification. NQI03-like eligibility features were evaluated only as sensitivity analyses. A standalone neonatal-age sensitivity restricted the cohort to discharges satisfying AGE = 0 and AGE_NEONATE = 1 without imposing an LOS restriction; the neonatal-age and LOS ≥ 3-day restrictions were also examined jointly in a separate mortality sensitivity analysis.

### 2.3. Exposure

The exposure was NQI03 numerator-code-positive bloodstream infection (NBSI), derived directly from the 2024 NQI03 technical specification [5]. Secondary diagnosis fields only (I10_DX2-I10_DX40) were searched. A discharge was positive if any BSI5DX code was present or if at least one BSI2DX code and at least one BSI3DX code were both present. Exact code sets are listed in Appendix B. Because the independently constructed cohort does not reproduce the full NQI03 denominator and diagnosis-level present-on-admission timing is unavailable, this exposure is not described as an exact NQI03 event, microbiologically confirmed sepsis, hospital-acquired infection, or preventable infection.

### 2.4. Outcomes

In-hospital mortality was defined by DIED = 1. Twelve cohort records had missing vital status and were not assigned an outcome value.

Mechanical ventilation was identified in I10_PR1-I10_PR25 using the AHRQ ICD-10-PCS codes 5A1935Z, 5A1945Z, and 5A1955Z [5]. Procedure timing relative to NBSI remains unknown. Mechanical ventilation was treated as a prespecified discharge-level clinical endpoint for analytic purposes. This terminology does not imply that ventilation occurred after NBSI; ventilation could have preceded, coincided with, or followed NBSI coding within the hospitalization.

Prolonged hospitalization was defined before complete-case exclusions from the unweighted LOS distribution in the independent cohort. PROC UNIVARIATE was used with the SAS default percentile definition (PCTLDEF = 5). The 85th, 90th, and 95th percentiles were 95, 108, and 130 days, respectively; the primary binary LOS outcome was therefore LOS ≥ 108 days. Analyses at 110 days, among survivors, and with log(LOS + 1) were used as sensitivities.

### 2.5. Covariates

The prespecified adjustment set emphasized baseline or structural characteristics that could be represented consistently in KID: birth-weight category (reference 1250–1499 g), completed gestational week and its quadratic term, sex, race/ethnicity, expected payer, transfer from another acute-care hospital, discharge quarter, hospital ownership, hospital location/teaching status, hospital bed size, and census region. Completed gestational week was derived from specific P07.2x/P07.3x codes; P07.21 (<23 completed weeks) was not forced to an exact week in continuous-age models. Race/ethnicity was grouped as White, Black, Hispanic, Asian/Pacific Islander, and Native American/Other; payer was grouped as Medicaid, private, self-pay, and other. Reference categories and source variables are listed in Appendix B. The target estimand was the adjusted contemporaneous discharge-level association between NBSI coding and each outcome conditional on these measured baseline and structural covariates; it was not interpreted as a causal effect.

Acute organ dysfunction and chronic comorbidity diagnosis counts were not included in the primary adjustment set because their timing relative to NBSI cannot be established and they may represent post-exposure conditions. P05 fetal growth/fetal malnutrition coding and a broad congenital Q-code flag were evaluated only as administrative sensitivity variables and were not interpreted as true small-for-gestational-age status or a validated major congenital anomaly measure.

### 2.6. Missing Data

Missingness was concentrated in race/ethnicity (4509; 11.38%) and completed gestational age (1540; 3.89%). Sex was missing in 28 records, expected payer in 66, discharge quarter in 8, mortality in 12, and LOS in 48; hospital design variables were complete. Missingness by NBSI and outcome status is summarized in Appendix B.

Fifty imputations were generated by fully conditional specification for incomplete baseline covariates, with predictive mean matching for gestational age and appropriate logistic or discriminant models for categorical variables [10,11]. For the primary mortality aOR, the 12 records with missing DIED were excluded before imputation, and observed mortality was included as a predictor in the imputation system. In the combined imputation system used for adjusted RRs and the non-mortality aORs, observed outcomes were included as auxiliary predictors; DIED and prolonged-LOS status could be imputed only to support covariate imputation, while each outcome-specific analysis was restricted to records with that outcome observed. Thus, no imputed outcome value contributed to its own analysis. Mechanical ventilation had no missing outcome values. Rubin’s rules were used to pool coefficients and standard errors. Complete-case models were retained as sensitivity analyses.

### 2.7. Statistical Analysis

Within each imputed dataset, binary outcomes were modeled with hospital-clustered logistic regression using HOSP_KID as the cluster; coefficients and standard errors were pooled with Rubin’s rules to obtain adjusted odds ratios. For clinical interpretability, adjusted risk ratios were estimated with modified-Poisson GEE models using empirical hospital-clustered standard errors. Adjusted absolute outcome probabilities were estimated in complete cases by record-level standardization (g-computation): each analytic record was scored twice after setting NBSI to 0 and 1 while all other covariates remained at their observed values, and predicted probabilities were averaged across the analytic sample. Risk differences were calculated from these standardized means. Multicollinearity was screened from the predictor design matrix using VIFs and condition indices. Observation-level influence statistics were obtained from corresponding ordinary logistic models solely as diagnostic screens; inferential estimates remained hospital-clustered, and no observations were removed post hoc on the basis of these diagnostics. Multiple imputation was performed with PROC MI using fully conditional specification; within-imputation clustered logistic models were fit with PROC SURVEYLOGISTIC and pooled with PROC MIANALYZE. Adjusted RRs were estimated with modified-Poisson PROC GENMOD models using a log link and REPEATED SUBJECT = HOSP_KID/TYPE = IND, with coefficients pooled using PROC MIANALYZE. Complete-case standardized probabilities were generated from PROC GENMOD models and record-level predictions obtained with PROC PLM SCORE, implementing marginal standardization as described previously [12,13]. Percentile 95% CIs for the standardized probabilities and risk differences were obtained from 1000 non-parametric hospital-cluster bootstrap samples generated with PROC SURVEYSELECT and refit with PROC LOGISTIC. Full-KID survey-domain sensitivities used PROC SURVEYLOGISTIC with DISCWT as the weight, KID_STRATUM as the stratum, HOSP_KID as the cluster, and DOMAIN analysis.

Full-KID survey-design sensitivities retained the complete KID and used domain analysis with DISCWT as the discharge weight, KID_STRATUM as the stratum, and HOSP_KID as the cluster. Additional analyses restricted the cohort to in-hospital births, non-transfers, or both; evaluated a standalone neonatal-age restriction (AGE = 0 and AGE_NEONATE = 1) for each endpoint without an LOS restriction; separately imposed LOS ≥ 3 days and the combined neonatal-age-plus-LOS restriction for mortality; used alternative LOS definitions; and refit models without the gestational age quadratic term and with a categorical gestational age specification. Hospital, payer, and race/ethnicity contrasts were exploratory and were not subjected to a multiplicity correction; they are interpreted using estimates and confidence intervals rather than isolated *p* values. The non-data-bearing SAS analysis programs (File S1) and the completed STROBE/RECORD reporting checklist (File S2) are provided as Supplementary Materials.

## 3. Results

### 3.1. Cohort and Crude Outcomes

**Figure 1:**
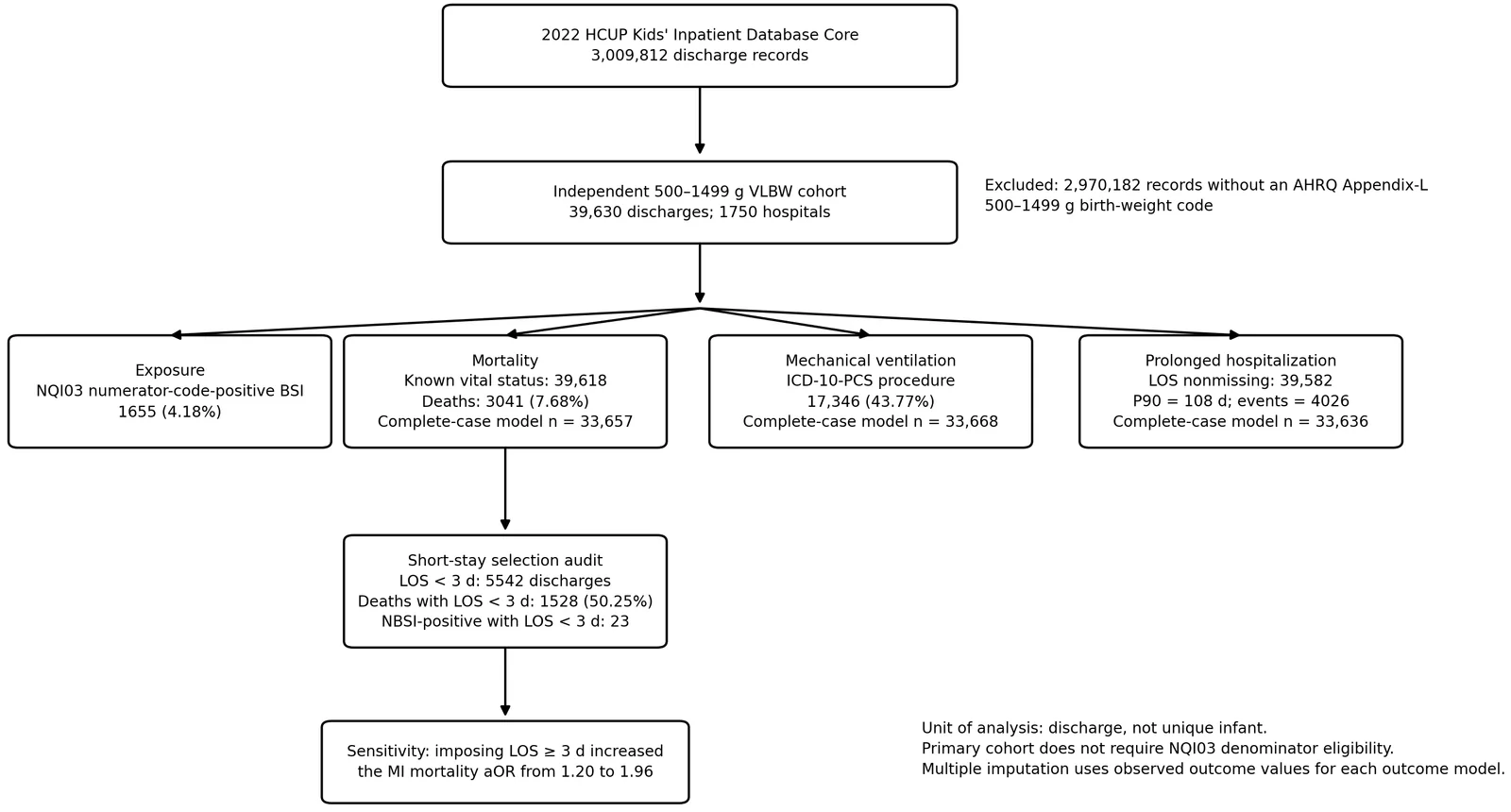
Cohort construction, exposure, outcomes, and the short-stay sensitivity. The primary cohort was constructed independently of the NQI03 denominator. BSI, bloodstream infection; LOS, length of stay; MI, multiple imputation; P90, 90th percentile.

The independent cohort contained 39,630 discharges from 1750 hospitals (Figure 1). NBSI was present in 1655 discharges (4.18%). Vital status was known for 39,618 discharges, among which 3041 deaths occurred (7.68%). Procedure-defined mechanical ventilation occurred in 17,346 discharges (43.77%), and 4026 of 39,582 discharges with nonmissing LOS met the 108-day prolonged-hospitalization threshold. Of all deaths, 1528 (50.25%) occurred during stays shorter than three days; only 23 NBSI-positive discharges had LOS < 3 days.

Crude mortality was 15.24% among NBSI-positive versus 7.35% among NBSI-negative discharges. Crude mechanical ventilation frequencies were 76.31% versus 42.35%, and crude prolonged-LOS frequencies were 28.94% versus 9.36%, respectively. Key characteristics of the cohort are summarized in Table 1.

**Table 1:** Key characteristics of the independent 500–1499 g cohort.

Characteristic	*n*/Denominator	Percent or Summary
Discharges	39,630	-
Hospitals	1750	-
Birth weight 500–749 g	7571/39,630	19.1%
Birth weight 750–999 g	8742/39,630	22.1%
Birth weight 1000–1249 g	10,240/39,630	25.8%
Birth weight 1250–1499 g	13,077/39,630	33.0%
Exact completed gestational week available	38,090/39,630	96.1%
Completed gestational age, median (IQR)	38,090 observed	28 weeks (26–30)
Neonatal-age proxy (AGE = 0 and AGE_NEONATE = 1)	36,722/39,630	92.66%
In-hospital birth	30,217/39,630	76.2%
Non-transfer	32,344/39,630	81.6%
NBSI	1655/39,630	4.18%
In-hospital mortality	3041/39,618	7.68%
Mechanical ventilation	17,346/39,630	43.77%
LOS ≥ 108 days	4026/39,582	10.17%
Deaths with LOS < 3 days	1528/3041 deaths	50.25%

Values are unweighted discharge counts. NBSI denotes NQI03 numerator-code-positive bloodstream infection.

### 3.2. Primary Adjusted Associations

After multiple imputation for missing baseline covariates, the NBSI mortality aOR was 1.200 (95% CI 1.005–1.432; *p* = 0.0434), with an adjusted RR of 1.165 (1.019–1.332). Associations were larger for mechanical ventilation (MI aOR 2.503, 95% CI 2.140–2.929; adjusted RR 1.290, 95% CI 1.240–1.342) and prolonged hospitalization (MI aOR 2.016, 95% CI 1.748–2.325; adjusted RR 1.597, 95% CI 1.454–1.754); both *p* values were <0.001. For mechanical ventilation, multiple imputation increased the point estimate from the complete-case aOR of 2.256 (1.926–2.643) to 2.503, while the direction and substantive conclusion were unchanged. These estimates are summarized in Table 2.

**Table 2:** Principal multiply imputed adjusted associations between NBSI and hospital outcomes.

Outcome	Analysis N	Events	MI aOR (95% CI)	MI Adjusted RR (95% CI)	*p* for aOR
In-hospital mortality	39,618	3041	1.200 (1.005–1.432)	1.165 (1.019–1.332)	0.0434
Mechanical ventilation	39,630	17,346	2.503 (2.140–2.929)	1.290 (1.240–1.342)	<0.0001
LOS ≥ 108 days	39,582	4026	2.016 (1.748–2.325)	1.597 (1.454–1.754)	<0.0001

Models adjust for birth-weight category, completed gestational week and its quadratic term, sex, race/ethnicity, payer, acute-care transfer, discharge quarter, hospital ownership, location/teaching status, bed size, and region. Adjusted RRs are from multiply imputed modified-Poisson GEE models with hospital-clustered empirical standard errors.

### 3.3. Secondary Complete-Case Adjusted Absolute Outcome Probabilities

Record-level standardization provided a secondary complete-case estimate of the corresponding absolute scale. For mortality, standardized probabilities were 7.5% under NBSI-positive versus 6.5% under NBSI-negative, an adjusted risk difference of 1.0 percentage point (95% bootstrap CI −0.3 to 2.2). For mechanical ventilation, the corresponding probabilities were 58.6% versus 42.6%, with a risk difference of 15.9 percentage points (95% CI 12.8–18.9). For prolonged hospitalization, probabilities were 15.4% versus 9.5%, with a risk difference of 5.9 percentage points (95% CI 4.4–7.5) (Table 3). These complete-case estimates complement, rather than replace, the multiply imputed aOR and adjusted-RR analyses.

### 3.4. Sensitivity and Model-Stability Analyses

The mortality point estimate was similar in complete cases (aOR 1.189, 95% CI 0.980–1.444) and in the full-KID survey-domain analysis (aOR 1.202, 95% CI 0.989–1.459). In contrast, imposing LOS ≥ 3 days increased the multiply imputed mortality aOR to 1.960 (95% CI 1.636–2.348), and additionally imposing the neonatal-age restriction within the LOS ≥ 3-day sensitivity yielded 1.666 (1.389–1.999). Sequential complete-case models showed the crude NBSI mortality OR of 2.272 attenuated to 1.349 after birth-weight adjustment and to 1.161 after gestational-age adjustment. Restricting the mortality model to in-hospital births (aOR 1.084), non-transfers (1.160), or both (1.090), and refitting it with a categorical gestational-age specification that included an unknown-gestational-age category (1.132), produced point estimates close to the complete-case estimate with confidence intervals spanning 1.0.

The standalone neonatal-age proxy (AGE = 0 and AGE_NEONATE = 1) was satisfied by 36,722 of 39,630 discharges (92.66%). Without imposing an LOS restriction, neonatal-age-restricted MI analyses yielded an aOR of 1.005 (95% CI 0.844–1.197; adjusted RR 1.012, 95% CI 0.890–1.151) for mortality, 2.093 (1.804–2.428; adjusted RR 1.200, 1.162–1.238) for mechanical ventilation, and 1.759 (1.541–2.007; adjusted RR 1.432, 1.320–1.553) for prolonged hospitalization. Thus, the mortality association attenuated to the null under the neonatal-age restriction, whereas the mechanical ventilation and prolonged-LOS associations remained positive.

Transfer and birth-location restrictions attenuated the associations modestly but left them clearly positive. Mechanical-ventilation aORs were 1.872 among in-hospital births, 2.065 among non-transfers, and 1.873 among discharges meeting both restrictions. Corresponding prolonged-LOS aORs were 1.764, 1.861, and 1.762. Removing the gestational-age quadratic term changed the NBSI estimates minimally for mechanical ventilation (2.256 to 2.245) and prolonged LOS (1.960 to 1.958); the mortality estimate changed from 1.189 to 1.223. Detailed sensitivity results are provided in Table A3.

### 3.5. Exploratory Patient and Hospital Associations

Because the principal objective concerned NBSI, secondary covariate findings are summarized rather than exhaustively interpreted. Birth weight and gestational age remained major maturity-related covariates. The positive quadratic gestational-age term in the mortality model indicated nonlinearity within the fixed VLBW range; because true small-for-gestational-age status was unavailable, no mechanistic interpretation was assigned to this pattern. Race/ethnicity, payer, and hospital contrasts were treated as exploratory descriptive associations rather than protective or harmful effects. Hospital-category outcome counts are provided in Table A5, including the sparse rural prolonged-LOS stratum, and model diagnostics are summarized in Table A6.

## 4. Discussion

### 4.1. Principal Findings

In an independently constructed VLBW cohort, NQI03 numerator-code-positive bloodstream infection showed three distinct patterns. The mortality association was modest after adjustment for neonatal maturity and baseline/structural covariates. In contrast, associations with procedure-defined mechanical ventilation and prolonged hospitalization were larger and remained evident across missing-data, transfer/inborn, survey-design, and model-specification sensitivities. Secondary complete-case standardized absolute differences were approximately 1.0 percentage point for mortality, 15.9 points for ventilation, and 5.9 points for prolonged hospitalization; the hospital-cluster bootstrap CI crossed zero for the mortality risk difference but not for ventilation or prolonged LOS.

The mortality result warrants particular restraint. Multiple imputation yielded an aOR of 1.20 with a lower confidence bound just above 1.0, whereas the complete-case and survey-domain confidence intervals crossed 1.0. The point estimates were similar across these specifications, indicating a modest association with uncertainty around the null rather than a qualitatively different effect. Consistent with this uncertainty, the standalone neonatal-age-restricted MI analysis yielded a mortality aOR of 1.005 (95% CI 0.844–1.197).

### 4.2. Comparison with Previous Work

Our 2019 KID study examined factors associated with the occurrence of NBSI [6], whereas the present analysis evaluates discharge outcomes associated with an NQI03 numerator-code construct in an independently selected VLBW cohort. Clinical studies use substantially richer infection definitions and physiologic information. For example, Fleiss and colleagues studied 653 preterm VLBW infants with confirmed late-onset infection across seven NICUs and reported 97 infection-episode deaths (15%) [4]. Crude in-hospital mortality among NBSI-positive discharges in the present cohort was also approximately 15%, but the quantities are not equivalent: the clinical study anchored mortality to a confirmed infection episode, whereas KID provides an administrative code-positive exposure and all-cause in-hospital death with unknown temporal ordering. Likewise, published population incidence estimates for neonatal sepsis are not directly comparable with the 4.18% code-positive prevalence observed here because the present denominator is a selected VLBW discharge cohort rather than live births [1,2]. These definitional and denominator differences are central when comparing administrative and clinical estimates.

### 4.3. Methodological Implications of the NQI03 Denominator

The short-stay analysis is the clearest methodological finding. More than half of the VLBW deaths occurred during stays shorter than three days, whereas only 1.39% of NBSI-positive discharges had LOS < 3 days. Imposing the NQI03-like short-stay rule therefore changed the mortality association far more than survey weighting or multiple imputation. This does not identify a formal causal bias direction, but it demonstrates that a quality indicator denominator designed for surveillance can materially change a patient-level outcome comparison when reused as an analytic cohort.

For clinicians and quality teams, NQI03 should therefore be interpreted primarily as a system-level surveillance and quality improvement signal rather than as an individual-level causal or prognostic measure. Changes in the indicator should prompt review of case mix, coding, infection-prevention processes, and relevant clinical events in context. Denominator rules designed for standardized benchmarking should not automatically be repurposed as patient-level eligibility criteria for etiologic or outcome research.

### 4.4. Mechanical Ventilation and Prolonged Hospitalization

Mechanical ventilation was ascertained with exact AHRQ ICD-10-PCS procedure codes. The NBSI association was similar under survey weighting and remained positive after in-hospital-birth and non-transfer restrictions, although the restricted estimates were modestly attenuated. Procedure timing relative to NBSI is unknown, so the analysis cannot establish whether infection preceded ventilation. Accordingly, the observed association should be interpreted as contemporaneous co-occurrence at the discharge level rather than evidence that NBSI caused subsequent ventilatory support.

The prolonged-hospitalization association was also robust, but temporal interpretation is even more constrained. Infection may prolong hospitalization, while longer hospitalization also creates greater opportunity for infection to occur, be detected, and be coded. The 108-day threshold is cohort-specific and dichotomizes a highly skewed variable; alternative thresholds and continuous LOS analyses support robustness of association but do not resolve directionality. Neonatal LOS is strongly influenced by maturity and clinical complexity, and prior neonatal studies have emphasized both its marked variability and the importance of birth weight and gestational age in its interpretation [14,15]. In the continuous model, exp(beta) = 2.036 describes the geometric-mean ratio for LOS + 1 and is not directly comparable with the adjusted RR for crossing the 108-day threshold because the two measures summarize different scales and portions of the LOS distribution.

### 4.5. Patient and Hospital Characteristics

Birth weight showed the strongest observed mortality gradient, and the sequential models demonstrate that birth weight and gestational age account for much of the crude NBSI-mortality association. The non-linear gestational age term was not interpreted mechanistically because true small-for-gestational-age status was unavailable. Exploratory payer, race/ethnicity, and hospital coefficients should not be interpreted as causal disparities or institutional performance measures. In particular, the rural prolonged-LOS estimate was based on only 16 events among 899 rural discharges and is vulnerable to sparse-cell instability. KID also lacks NICU level, neonatal volume, staffing, referral intensity, and other structural variables needed for causal institutional comparisons [16,17].

### 4.6. Strengths

Strengths include a large contemporary multi-hospital VLBW cohort, outcome-independent cohort construction, direct adjustment for completed gestational age, exact procedure-code ascertainment of mechanical ventilation, separate modeling of clinically distinct outcomes, multiple imputation for baseline missingness, full-KID survey-design sensitivity, adjusted absolute outcome probabilities, and targeted transfer/inborn and model-stability analyses. The explicit audit of the NQI03 denominator and the separation of indicator numerator logic from cohort eligibility are additional methodological strengths.

### 4.7. Limitations

The principal limitation is temporal ambiguity. NBSI and the outcomes are recorded within the same hospitalization, and infection onset is unavailable. None of the associations can establish that NBSI preceded or caused an outcome. This limitation is particularly important for LOS because of time-at-risk and reverse-causation mechanisms. For the same reason, time-to-event or Cox proportional-hazards models with NBSI as a time-varying exposure could not be validly implemented: KID does not provide infection-onset time or the event-time information needed to order NBSI relative to mechanical ventilation, death, or discharge.

Second, NBSI is an administrative coding construct. The data cannot distinguish early- from late-onset infection, central-line-associated infection, hospital-acquired or preventable events, or microbiologically confirmed infection. Apgar scores and microbiology were unavailable. P05 coding is not a true small-for-gestational-age measure, and broad congenital Q-codes do not constitute a validated major-anomaly severity algorithm.

Third, the unit of analysis is a discharge; repeated admissions cannot be linked, and outcomes end at discharge. Important residual confounders are unavailable, including detailed congenital anomaly severity, antenatal exposures, central-line exposure, NICU level, neonatal volume, staffing, referral intensity, feeding progression, and richer socioeconomic measures. Multiple imputation addresses missing observed covariates under its assumptions but cannot correct unmeasured confounding.

Fourth, the 108-day LOS threshold is cohort-derived and may not generalize to other populations. Survivor restriction does not solve competing-risk bias and was therefore treated only as a sensitivity analysis. Finally, the data are from one US administrative database and one year; coding practices and neonatal care systems may differ elsewhere.

## 5. Conclusions

Among 2022 KID discharges weighing 500–1499 g, NQI03 numerator-code-positive bloodstream infection was associated with more pronounced adjusted differences in mechanical ventilation and prolonged hospitalization and with a modest, imprecisely estimated difference in in-hospital mortality. Secondary complete-case standardized absolute differences were approximately 15.9 percentage points for mechanical ventilation, 5.9 percentage points for prolonged hospitalization, and 1.0 percentage point for mortality.

Mortality estimates were much more sensitive to the NQI03 short-stay criterion than to survey weighting or missing-data handling. Quality indicator eligibility rules should therefore be examined before they are reused for patient-level outcome analyses. Because infection timing is unavailable, the findings remain contemporaneous discharge-level associations rather than causal effects.

## Figures and Tables

**Table 3:** Secondary complete-case record-level standardized probabilities and adjusted absolute differences associated with NQI03 numerator-code-positive bloodstream infection.

Outcome	Standardized Probability, NBSI-Negative (95% CI)	Standardized Probability, NBSI-Positive (95% CI)	Adjusted Risk Difference, Percentage Points (95% CI)
Mortality	6.5% (6.2–6.9)	7.5% (6.2–8.8)	+1.0 (−0.3 to 2.2)
Mechanical ventilation	42.6% (41.2–44.0)	58.6% (55.2–62.1)	+15.9 (12.8–18.9)
LOS ≥ 108 days	9.5% (8.8–10.2)	15.4% (13.7–17.2)	+5.9 (4.4–7.5)

Probabilities were standardized over each outcome’s complete-case analytic sample by record-level g-computation. Every record was scored under NBSI = 0 and NBSI = 1 with all other covariates held at their observed values; displayed probabilities are the averages of those standardized predictions. Risk differences are NBSI-positive minus NBSI-negative. Analytic N was 33,657 for mortality, 33,668 for mechanical ventilation, and 33,636 for prolonged LOS. These are secondary complete-case standardized estimates. Percentile 95% CIs were obtained from 1000 non-parametric hospital-cluster bootstrap replicates; the multiply imputed aORs and adjusted RRs in Table 2 remain the principal inferential estimates.

## Data Availability

The 2022 KID is available for purchase from the Agency for Healthcare Research and Quality Healthcare Cost and Utilization Project under its data-use agreement. The authors do not have permission to redistribute the underlying data. The variable dictionary is provided in Appendix B. Non-data-bearing SAS programs and the completed STROBE/RECORD checklist accompany the submission as Supplementary Materials and are also available from the corresponding author.

## Supplementary Materials

The following supporting information can be downloaded at: https://www.mdpi.com/article/10.3390/children13091182/s1, The following files accompany the submission: File S1, non-data-bearing SAS analysis programs; File S2, completed STROBE/RECORD reporting checklist.

## Abbreviations

AHRQ	Agency for Healthcare Research and Quality
aOR	adjusted odds ratio
CI	confidence interval
FCS	fully conditional specification
GEE	generalized estimating equation
HCUP	Healthcare Cost and Utilization Project
KID	Kids’ Inpatient Database
LOS	length of stay
MI	multiple imputation
NBSI	NQI03 numerator-code-positive bloodstream infection
NQI03	Neonatal Quality Indicator 03
P90	90th percentile
PCS	Procedure Coding System
RR	risk ratio
SGA	small for gestational age
VLBW	very low birth weight

## Appendix A. Sensitivity Analyses and Full Model Results

### Appendix A.1. Cohort and Short-Stay Audit

**Table A1:** Cohort and short-stay audit.

Item	Result	Interpretation
Independent VLBW cohort	39,630 discharges; 1750 hospitals	Primary analytic population
NBSI positive	1655 (4.18%)	Exposure
LOS < 3 days	5542 discharges	Retained in primary cohort
Deaths with LOS < 3 days	1528 (50.25% of deaths)	Short-stay selection concern
NBSI-positive with LOS < 3 days	23 (1.39% of NBSI)	Few exposure-positive records removed by rule
Exact completed week	38,090 (96.1%)	Supports GA adjustment
P90 LOS	108 days	Primary prolonged-LOS threshold

### Appendix A.2. Mortality Attenuation and Selection

**Table A2:** Mortality attenuation and selection.

Model	Adjustment/Restriction	aOR	95% CI
A	NBSI only	2.272	1.915–2.696
B	+birth-weight category	1.349	1.128–1.614
C	+completed gestational age	1.161	0.960–1.403
D	+patient/discharge covariates	1.197	0.989–1.450
E	+hospital covariates	1.189	0.980–1.444
F	Model E + LOS ≥ 3 days	1.787	1.446–2.210
G	Model E + neonatal-age proxy + LOS ≥ 3	1.542	1.240–1.918
H1	Model E + P05 coding	1.188	0.979–1.442
H2	Model E + any congenital Q-code	1.282	1.058–1.553
H3	Model E + P05 + any Q-code	1.280	1.057–1.551
I	Missing-category sensitivity	1.237	1.041–1.471

P05 and broad Q-code flags are administrative coding sensitivities, not validated SGA or major congenital anomaly measures.

### Appendix A.3. Principal NBSI Sensitivity Estimates

**Table A3:** Principal NBSI sensitivity estimates.

Outcome/Sensitivity	Estimate	95% CI/Note
Mortality, MI primary	1.200	1.005–1.432
Mortality, complete case	1.189	0.980–1.444
Mortality, full-KID survey domain	1.202	0.989–1.459
Mortality, GA categorical sensitivity	1.132	0.946–1.355; includes unknown GA category
Mortality, in-hospital births	1.084	0.875–1.343; complete case
Mortality, non-transfers	1.160	0.942–1.427; complete case
Mortality, inborn + non-transfer	1.090	0.879–1.350; complete case
Mortality, MI + LOS ≥ 3	1.960	1.636–2.348
Mortality, MI neonatal-age proxy only	1.005	0.844–1.197; adjusted RR 1.012 (0.890–1.151)
Mortality, MI + neonatal-age proxy + LOS ≥ 3	1.666	1.389–1.999
Mechanical ventilation, MI	2.503	2.140–2.929
Mechanical ventilation, complete case	2.256	1.926–2.643
Mechanical ventilation, survey domain	2.278	1.947–2.664
Mechanical ventilation, in-hospital births	1.872	1.578–2.221
Mechanical ventilation, non-transfers	2.065	1.749–2.439
Mechanical ventilation, inborn + non-transfer	1.873	1.579–2.223
Mechanical ventilation, MI neonatal-age proxy only	2.093	1.804–2.428; adjusted RR 1.200 (1.162–1.238)
LOS ≥ 108 d, MI	2.016	1.748–2.325
LOS ≥ 108 d, complete case	1.960	1.697–2.263
LOS ≥ 108 d, survey domain	1.949	1.688–2.252
LOS ≥ 108 d, in-hospital births	1.764	1.497–2.079
LOS ≥ 108 d, non-transfers	1.861	1.586–2.183
LOS ≥ 108 d, inborn + non-transfer	1.762	1.494–2.078
LOS ≥ 108 d, MI neonatal-age proxy only	1.759	1.541–2.007; adjusted RR 1.432 (1.320–1.553)
LOS ≥ 110 d, complete case	1.907	1.647–2.207
LOS ≥ 110 d, survivors	1.961	1.674–2.296
Continuous log(LOS + 1), hospital-clustered GEE	+0.7111 (SE 0.0404)	95% CI 0.6319–0.7903; *p* < 0.0001; exp(beta) = 2.036 on the geometric scale

### Appendix A.4. Complete-Case Regression Coefficients and Global Tests

**Table A4:** Complete-case regression coefficients and global tests.

Predictor/Contrast	Mortality aOR (95% CI)	Mechanical Ventilation aOR (95% CI)	LOS ≥ 108 d aOR (95% CI)
NBSI	1.189 (0.980–1.444)	2.256 (1.926–2.643)	1.960 (1.697–2.263)
BW 500–749 vs. 1250–1499 g	3.765 (2.942–4.818)	1.695 (1.517–1.894)	6.650 (5.305–8.336)
BW 750–999 vs. 1250–1499 g	2.136 (1.715–2.661)	1.427 (1.309–1.556)	4.263 (3.455–5.261)
BW 1000–1249 vs. 1250–1499 g	1.371 (1.120–1.677)	1.128 (1.052–1.210)	1.816 (1.473–2.240)
Gestational age, centered linear	0.950 (0.917–0.984)	0.705 (0.689–0.721)	0.693 (0.643–0.747)
Gestational age squared	1.033 (1.027–1.038)	0.988 (0.984–0.993)	0.985 (0.975–0.994)
Male vs. female	1.344 (1.222–1.478)	1.191 (1.135–1.250)	1.121 (1.033–1.216)
Black vs. White	0.806 (0.709–0.917)	0.917 (0.838–1.003)	0.847 (0.747–0.960)
Hispanic vs. White	0.926 (0.806–1.065)	0.845 (0.764–0.935)	0.941 (0.807–1.099)
Asian/PI vs. White	0.805 (0.614–1.055)	0.826 (0.708–0.964)	1.191 (0.944–1.502)
Native American/Other vs. White	1.375 (1.149–1.646)	0.820 (0.721–0.931)	0.849 (0.704–1.023)
Medicaid vs. private	0.976 (0.870–1.095)	1.044 (0.979–1.114)	1.057 (0.956–1.169)
Self-pay vs. private	3.330 (2.569–4.315)	0.842 (0.695–1.021)	0.328 (0.218–0.494)
Other payer vs. private	0.752 (0.596–0.948)	0.983 (0.834–1.158)	1.016 (0.834–1.238)
Acute transfer vs. no	1.144 (0.992–1.320)	1.103 (0.939–1.295)	1.555 (1.297–1.865)
Q2 vs. Q1	1.034 (0.899–1.188)	0.982 (0.911–1.058)	0.924 (0.819–1.042)
Q3 vs. Q1	1.018 (0.895–1.158)	0.988 (0.915–1.067)	0.913 (0.806–1.034)
Q4 vs. Q1	0.944 (0.822–1.085)	0.952 (0.883–1.027)	0.925 (0.820–1.043)
Government vs. private non-profit	1.116 (0.953–1.307)	1.095 (0.896–1.338)	1.058 (0.836–1.340)
Investor-owned vs. private non-profit	1.286 (1.054–1.570)	0.871 (0.644–1.179)	0.644 (0.459–0.902)
Rural vs. urban teaching	0.588 (0.400–0.865)	0.724 (0.555–0.944)	0.157 (0.055–0.450)
Urban non-teaching vs. urban teaching	0.796 (0.634–1.000)	0.963 (0.806–1.149)	0.363 (0.242–0.544)
Small vs. large bed size	0.838 (0.686–1.024)	0.833 (0.658–1.053)	0.583 (0.445–0.764)
Medium vs. large bed size	0.929 (0.804–1.072)	0.926 (0.792–1.082)	0.667 (0.523–0.851)
Northeast vs. West	0.826 (0.680–1.004)	0.954 (0.790–1.151)	1.310 (1.025–1.674)
Midwest vs. West	1.000 (0.814–1.227)	1.160 (0.987–1.363)	1.425 (1.089–1.863)
South vs. West	1.040 (0.890–1.216)	0.902 (0.771–1.055)	1.424 (1.147–1.769)

Global Type 3 *p* values: mortality—NBSI 0.0800, birth weight < 0.0001, gestational age linear 0.0039, gestational age quadratic < 0.0001, sex < 0.0001, race/ethnicity < 0.0001, payer < 0.0001, transfer 0.0643, quarter 0.5006, ownership 0.0280, location/teaching 0.0051, bed size 0.1754, region 0.0638. Mechanical ventilation—NBSI < 0.0001, birth weight < 0.0001, gestational age terms < 0.0001, sex < 0.0001, race/ethnicity 0.0011, payer 0.1152, transfer 0.2337, quarter 0.6028, ownership 0.4352, location/teaching 0.0577, bed size 0.2486, region 0.0246. Prolonged LOS—NBSI < 0.0001, birth weight < 0.0001, gestational age linear < 0.0001, quadratic 0.0018, sex 0.0062, race/ethnicity 0.0128, payer < 0.0001, transfer < 0.0001, quarter 0.4592, ownership 0.0288, location/teaching < 0.0001, bed size < 0.0001, region 0.0107.

### Appendix A.5. Hospital-Category Counts and Outcome Events

**Table A5:** Hospital-category counts and outcome events.

Hospital Characteristic/Category	Hospitals	Discharges	Deaths	Mechanical Ventilation Events	LOS ≥ 108-Day Events
Ownership: Government/public	215	4486	366	2036	508
Ownership: Private, not-for-profit	1287	31,428	2360	13,858	3288
Ownership: Private, investor-owned	248	3716	315	1452	230
Location/teaching: Rural	337	899	51	322	16
Location/teaching: Urban, non-teaching	336	2565	167	1005	90
Location/teaching: Urban, teaching	1077	36,166	2823	16,019	3920
Bed size: Small	453	4268	303	1776	351
Bed size: Medium	538	8393	613	3533	670
Bed size: Large	759	26,969	2125	12,037	3005
Region: Northeast	262	5930	391	2474	598
Region: Midwest	409	8387	649	3995	899
Region: South	698	17,849	1420	7615	1881
Region: West	381	7464	581	3262	648

Mortality was missing for 12 discharges and LOS for 48 discharges overall. Event counts are descriptive and do not imply independent hospital effects. The rural prolonged-LOS stratum contained 16 events among 899 discharges, so corresponding regression contrasts warrant particular caution regarding sparse-cell instability.

### Appendix A.6. Model Diagnostics

**Table A6:** Model diagnostics.

Diagnostic	Model	Result
Multicollinearity	Mortality design matrix	Maximum VIF 2.904; maximum focused |correlation| among centered GA, GA squared, and birth-weight indicators 0.63; maximum condition index 11.997
Influence screen	Mortality (*n* = 33,657)	Max leverage 0.013043; 4388 above 2p/n screen; max DIFCHISQ 88.6856 (1472 > 3.84); max DIFDEV 9.0167 (1195 > 3.84)
Influence screen	Mechanical ventilation (*n* = 33,668)	Max leverage 0.003959; 1857 above 2p/n screen; max DIFCHISQ 47.3072 (1134 > 3.84); max DIFDEV 7.7730 (462 > 3.84)
Influence screen	LOS ≥ 108 days (*n* = 33,636)	Max leverage 0.011927; 4977 above 2p/n screen; max DIFCHISQ 1416.5087 (1322 > 3.84); max DIFDEV 14.6115 (876 > 3.84)

VIF and condition-index results did not indicate problematic multicollinearity. Influence statistics were used as screening diagnostics rather than post hoc exclusion rules; no observations were deleted on this basis. The LOS screening model produced an extreme maximum DIFCHISQ of 1416.5, which was therefore treated as a flag for cautious interpretation rather than as a deletion criterion. Principal exposure estimates were evaluated through prespecified missing-data, survey-design, short-stay, transfer/inborn, gestational age, and LOS-specification sensitivities.

## Appendix B. Variable Dictionary, Coding, Missingness, and Supplemental Descriptives

### Appendix B.1. Variable Dictionary

**Table A7:** Variable dictionary.

Construct	HCUP/KID Source	Operationalization	Reference/Role
NBSI exposure	I10_DX2-I10_DX40	Published NQI03 secondary-diagnosis numerator logic; see B2	Absent
Birth weight	I10_DX1-I10_DX40	AHRQ Appendix-L categories 500–1499 g; lowest category if multiple	1250–1499 g
Gestational age	I10_DX1-I10_DX40	Specific P07.2x/P07.3x completed-week codes; centered week + squared term	Continuous
Mortality	DIED	1 = death during hospitalization	0 = survived
Mechanical ventilation	I10_PR1-I10_PR25	5A1935Z, 5A1945Z, 5A1955Z	Absent
LOS	LOS	Primary threshold ≥ 108 d; alternatives ≥ 110 d and log(LOS + 1)	Below threshold
Sex	FEMALE	0 = male; 1 = female	Female
Race/ethnicity	RACE	White; Black; Hispanic; Asian/PI; Native American + Other	White
Expected payer	PAY1	Medicaid; private; self-pay; other	Private
Acute transfer	TRAN_IN	1 = another acute-care hospital	No acute transfer
Quarter	DQTR	1–4	Q1
Ownership	H_CONTRL	Government; private non-profit; investor-owned	Private nonprofit
Location/teaching	HOSP_LOCTEACH	Rural; urban non-teaching; urban teaching	Urban teaching
Bed size	HOSP_BEDSIZE	Small; medium; large	Large
Region	HOSP_REGION	Northeast; Midwest; South; West	West
Survey design	DISCWT, KID_STRATUM, HOSP_KID	Weight, stratum, cluster	Sensitivity
In-hospital birth	I10_HOSPBRTH	1 = in-hospital birth	Sensitivity
Neonatal-age proxy	AGE, AGE_NEONATE	AGE = 0 and AGE_NEONATE = 1	Sensitivity

### Appendix B.2. Exact Code Sets

**Table A8:** Exact code sets.

Definition	Codes	Use
Birth weight 500–749 g	P0502; P0512; P0702	Cohort
Birth weight 750–999 g	P0503; P0513; P0703	Cohort
Birth weight 1000–1249 g	P0504; P0514; P0714	Cohort
Birth weight 1250–1499 g	P0505; P0515; P0715	Cohort
BSI5DX	P3619; P3639; P362; P364; P3630	NBSI if any secondary code
BSI2DX	P368; R7881	Requires secondary BSI3DX
BSI3DX	B961; B9620; B9621; B9622; B9623; B9629; B965; B9683; B9689	Requires secondary BSI2DX
Mechanical ventilation	5A1935Z; 5A1945Z; 5A1955Z	Outcome
Completed weeks 23–27	23 = P0722; 24 = P0723; 25 = P0724; 26 = P0725; 27 = P0726	GA adjustment
Completed weeks 28–36	28 = P0731; 29 = P0732; 30 = P0733; 31 = P0734; 32 = P0735; 33 = P0736; 34 = P0737; 35 = P0738; 36 = P0739	GA adjustment
<23 completed weeks	P0721	Categorical sensitivity only; not forced to exact week

### Appendix B.3. Missingness

**Table A9:** Missingness.

Variable	Observed *n*	Missing *n*	Missing %
NBSI exposure	39,630	0	0.00%
Mortality	39,618	12	0.03%
Mechanical ventilation	39,630	0	0.00%
LOS	39,582	48	0.12%
Birth weight	39,630	0	0.00%
Completed gestational week	38,090	1540	3.89%
Sex	39,602	28	0.07%
Race/ethnicity	35,121	4509	11.38%
Expected payer	39,564	66	0.17%
Discharge quarter	39,622	8	0.02%
Hospital design variables	39,630	0	0.00%

Among NBSI-negative versus NBSI-positive discharges, missing completed gestational age was 3.75% versus 7.01%, and missing race/ethnicity was 11.44% versus 9.91%. Missing gestational age was 3.09% without versus 4.91% with mechanical ventilation, and 3.26% without versus 9.17% with prolonged LOS.

### Appendix B.4. Modeled Covariate Distributions by NBSI Status

**Table A10:** Modeled covariate distributions by NBSI status.

Characteristic	NBSI-Negative (*n* = 37,975)	NBSI-Positive (*n* = 1655)
**Sex**		
Male	19,084 (50.25%)	927 (56.01%)
Female	18,864 (49.67%)	727 (43.93%)
Missing	27 (0.07%)	1 (0.06%)
**Race/ethnicity**		
White	12,907 (33.99%)	540 (32.63%)
Black	9569 (25.20%)	444 (26.83%)
Hispanic	6681 (17.59%)	312 (18.85%)
Asian/Pacific Islander	1450 (3.82%)	64 (3.87%)
Native American/Other	3023 (7.96%)	131 (7.92%)
Missing	4345 (11.44%)	164 (9.91%)
**Expected primary payer**		
Medicaid	20,975 (55.23%)	891 (53.84%)
Private	13,901 (36.61%)	640 (38.67%)
Self-pay	1161 (3.06%)	32 (1.93%)
Other	1873 (4.93%)	91 (5.50%)
Missing	65 (0.17%)	1 (0.06%)
**Discharge quarter**		
Q1	9244 (24.34%)	391 (23.63%)
Q2	9095 (23.95%)	390 (23.56%)
Q3	9805 (25.82%)	417 (25.20%)
Q4	9824 (25.87%)	456 (27.55%)
Missing	7 (0.02%)	1 (0.06%)
**Hospital ownership**		
Government/public	4270 (11.24%)	216 (13.05%)
Private, not-for-profit	30,117 (79.31%)	1311 (79.21%)
Private, investor-owned	3588 (9.45%)	128 (7.73%)
**Hospital location/teaching**		
Rural	880 (2.32%)	19 (1.15%)
Urban, non-teaching	2498 (6.58%)	67 (4.05%)
Urban, teaching	34,597 (91.10%)	1569 (94.80%)
**Hospital bed size**		
Small	4132 (10.88%)	136 (8.22%)
Medium	8065 (21.24%)	328 (19.82%)
Large	25,778 (67.88%)	1191 (71.96%)
**Hospital region**		
Northeast	5676 (14.95%)	254 (15.35%)
Midwest	8039 (21.17%)	348 (21.03%)
South	17,104 (45.04%)	745 (45.02%)
West	7156 (18.84%)	308 (18.61%)

Percentages use the full NBSI-group denominator. Missing categories are displayed explicitly for sex, race/ethnicity, payer, and discharge quarter; hospital variables were complete.
