# Supplementary material for: Bloodstream Infection and Hospital Outcomes Among Very Low Birth Weight Discharges: Mortality, Mechanical Ventilation, and Prolonged Hospitalization in the 2022 HCUP Kids’ Inpatient Database
Source: Children (Basel). 2026 Sep 2;13(9):1182. doi: 10.3390/children13091182 (PMC13605882; doi:10.3390/children13091182)
Supplement: Supplementary file 1 [file children-13-01182-s001.zip › File_S2_Completed_STROBE_RECORD_Checklist.pdf]

File S2. Completed STROBE / RECORD Reporting Checklist

Manuscript ID: children-4500895

Bloodstream Infection and Hospital Outcomes Among Very Low Birth Weight Discharges: Mortality, Mechanical Ventilation, and Prolonged Hospitalization in the 2022 HCUP Kids' Inpatient Database

This crosswalk uses the published STROBE item numbers and the 13 RECORD extension items. Requirement descriptions are concise paraphrases rather than reproductions of the published checklists. Page numbers refer to the accepted 18-page manuscript after the final round of revisions.

Item	Requirement (concise paraphrase)	Page(s)	How addressed
**STROBE 1**	Indicate the study design in the title/abstract and provide an informative summary.	1-3	The title and structured abstract identify the 2022 KID, VLBW discharge cohort, principal endpoints, adjusted estimates, and major limitations; the retrospective cross-sectional design is stated explicitly at the start of Methods 2.1 (p. 3).
**RECORD 1.1**	Name the type of routinely collected data and, where possible, the database in the title/abstract.	1-3	The HCUP Kids' Inpatient Database (KID) is named in the title and abstract. Its administrative, all-payer discharge-data nature is described in the abstract and Methods 2.1.
**RECORD 1.2**	Report the geographic region and time frame in the title/abstract when applicable.	1-3	The year 2022 is stated in the title and abstract; Methods 2.1 identifies KID as a US all-payer pediatric inpatient database and the conclusions limit inference to 2022 KID discharges.
**RECORD 1.3**	If linkage between databases was conducted, state this in the title/abstract.	3	No person-level or external-database linkage was performed. Hospital characteristics from the companion KID Hospital file were merged to the KID Core by HOSP_KID, as described in Methods 2.1.
**STROBE 2**	Explain the scientific background and rationale.	2	The Introduction summarizes neonatal bloodstream infection, limitations of administrative data, the NQI03 denominator issue, and the rationale for an outcome-independent VLBW cohort.
**STROBE 3**	State specific objectives and any prespecified hypotheses/aims.	2-3	The primary mortality objective, secondary mechanical-ventilation/prolonged-LOS objectives, robustness analyses, and exploratory patient/hospital contrasts are explicitly distinguished.
**STROBE 4**	Present key elements of the study design early.	3	Methods 2.1 identifies a retrospective cross-sectional, discharge-level analysis of the 2022 KID.
**STROBE 5**	Describe the setting, locations, and relevant dates.	3	Methods 2.1 describes the 2022 US all-payer pediatric inpatient setting, KID Core, and companion Hospital file.
**STROBE 6**	Describe record eligibility, selection, and follow-up/eligibility methods as applicable.	3, 6, 12	Methods 2.2 defines the independent 500-1,499 g cohort and explicitly omits NQI03 denominator restrictions; Figure 1/Table 1 and Appendix A1 quantify cohort selection and short-stay retention.
**RECORD 6.1**	List in detail the codes/algorithms used to select the study population, or explain why this is not possible.	3, 16	Methods 2.2 describes the AHRQ Appendix-L birth-weight algorithm and multiple-code rule; exact ICD-10-CM code sets are provided in Appendix B2.
**RECORD 6.2**	Reference validation studies of population-selection codes/algorithms, or describe validation performed for the study.	3, 16	Population identification uses published AHRQ Appendix-L code specifications. No separate external validation study of this custom VLBW cohort algorithm was performed or claimed.
**RECORD 6.3**	If database linkage was used, consider a flow diagram showing the linkage process and linked-record counts.	3, 6	No person-level/external-database linkage was performed. The internal KID Core-to-Hospital merge by HOSP_KID is described in Methods 2.1; Figure 1 displays cohort selection, and hospital design variables were complete in the analytic cohort.
**STROBE 7**	Define outcomes, exposures, predictors, potential confounders, and effect modifiers.	3-4, 15-16	Methods 2.3-2.5 and Appendix B1 define NBSI, mortality, mechanical ventilation, prolonged LOS, baseline covariates, hospital characteristics, and sensitivity variables.
**RECORD 7.1**	Provide a complete list of codes/algorithms used to classify exposures, outcomes, confounders, and effect modifiers.	3-4, 16	Appendix B2 provides the exact NQI03 numerator diagnosis sets, mechanical-ventilation PCS codes, birth-weight codes, and completed gestational-week mappings.
**STROBE 8**	Describe data sources and measurement/assessment methods.	3-4, 15-16	Methods and Appendix B describe HCUP Core/Hospital sources and operational definitions for the exposure, endpoints, gestational age, patient/discharge variables, hospital characteristics, and survey variables.
**STROBE 9**	Describe efforts to address potential sources of bias.	3-4, 8-10, 12-15	The analysis uses outcome-independent cohort construction, short-stay and standalone neonatal-age sensitivities, multiple imputation, survey-domain analysis, inborn/non-transfer restrictions, gestational-age specification checks, cluster-bootstrap uncertainty, and explicitly non-causal interpretation.
**STROBE 10**	Explain how the study size was determined.	3, 6	All KID discharges meeting the prespecified 500-1,499 g coding definition were included; no separate sample-size target was imposed.

Item	Requirement (concise paraphrase)	Page(s)	How addressed
**STROBE 11**	Explain handling of quantitative variables and any groupings.	4, 13	Birth weight is modeled categorically, gestational age continuously with a quadratic term, LOS P90 is derived by PROC UNIVARIATE, and alternative/continuous LOS specifications are reported in Appendix A3.
**STROBE 12**	Describe statistical methods, confounding control, missing-data handling, subgroup/sensitivity analyses, and other relevant analyses.	4-5, 8, 13-15	Methods 2.6-2.7 describe FCS multiple imputation, clustered logistic and modified-Poisson GEE models, Rubin pooling, complete-case record standardization, 1,000-replicate hospital-cluster bootstrap CIs, full-KID survey-domain sensitivity, neonatal-age and transfer/inborn restrictions, LOS alternatives, and multiplicity handling for exploratory contrasts.
**RECORD 12.1**	Describe the extent of investigators' access to the database used to create the study population.	3, 11	The study uses licensed HCUP KID files. The Data Availability Statement explains that the underlying data are available from HCUP under its data-use agreement and cannot be redistributed by the authors.
**RECORD 12.2**	Describe data-cleaning methods used in the study.	3-4, 15-17	Methods and Appendix B document diagnosis/procedure normalization, birth-weight recoding, gestational-age derivation, exposure/outcome coding, category collapsing, LOS thresholding/transformation, missingness, and reference categories.
**RECORD 12.3**	State whether person-level, institutional-level, or other linkage was used and describe linkage methods/quality as applicable.	3	No person-level or external-database linkage was used. Hospital characteristics from the KID Hospital file were merged to Core discharge records by HOSP_KID; hospital design variables were complete in the analytic cohort.
**STROBE 13**	Report numbers of records at each study stage and reasons for exclusion/non-participation.	6, 12	Figure 1 reports 3,009,812 Core records, 2,970,182 exclusions, the 39,630-discharge cohort, endpoint counts, short stays, and complete-case Ns; Appendix A1 supplements the flow.
**RECORD 13.1**	Describe study-population selection in detail, including filtering for data quality/availability/linkage.	3, 6, 12, 16	Methods, Figure 1, Appendix A1, and Appendix B2 document the selection logic and exact code sets; the primary cohort is explicitly independent of NQI03 denominator eligibility.
**STROBE 14**	Provide descriptive characteristics and report missingness for variables of interest.	6, 16-17	Table 1 reports key cohort characteristics; Appendix B3 reports variable-specific missingness and missingness by exposure/outcome; Appendix B4 reports modeled covariate distributions by NBSI status.
**STROBE 15**	Report numbers of outcome events or summary measures.	5-6, 14-15	Crude deaths, mechanical-ventilation events, and prolonged-LOS events are reported overall and by NBSI status; Appendix A5 reports hospital-category event counts.
**STROBE 16**	Report main adjusted results with precision and identify variables adjusted for.	7	Table 2 reports multiply imputed aORs and adjusted RRs with 95% CIs. Table 3 reports secondary complete-case standardized probabilities and risk differences with percentile 95% CIs; the adjustment set is specified in Methods and the Table 2 footnote.
**STROBE 17**	Report other analyses, including sensitivity and subgroup analyses.	8, 12-15	Short-stay, standalone neonatal-age, combined neonatal-age-plus-short-stay mortality, survey-domain, inborn/non-transfer, alternative LOS, continuous LOS, gestational-age specification, coding, diagnostic, and missing-data analyses are reported.
**STROBE 18**	Summarize key results with reference to study objectives.	8	Discussion 4.1 summarizes the modest/imprecise mortality association, larger ventilation/LOS associations, secondary absolute differences with bootstrap uncertainty, and attenuation of mortality in the standalone neonatal-age sensitivity.
**STROBE 19**	Discuss study limitations, including potential direction/magnitude of bias where possible.	10-11	Limitations address temporal ambiguity, inability to construct a valid time-varying NBSI exposure for Cox analysis, administrative coding, unavailable neonatal confounders, discharge-level inference, competing mortality, cohort-specific LOS, and database/year generalizability.
**RECORD 19.1**	Discuss implications of using routinely collected data not created for the specific research question.	10-11	The manuscript discusses absence of infection onset/event-time ordering, microbiology, Apgar, true SGA, detailed anomaly severity, NICU structural measures, post-discharge outcomes, and diagnosis-level POA timing, as well as residual confounding and misclassification.
**STROBE 20**	Provide a cautious overall interpretation considering objectives, limitations, multiplicity, and other evidence.	8-11	The Discussion separates clinical association from causal interpretation, compares administrative and clinical literature, and emphasizes uncertainty, time-at-risk, selection, and specification differences.
**STROBE 21**	Discuss the generalizability of the study results.	10-11	Inference is limited to 2022 US KID discharges in the prespecified 500-1,499 g cohort; findings are not generalized to all NQI03-eligible infants, unique infants, or other years/health systems.
**STROBE 22**	State funding sources and the role of funders.	11	The Funding statement reports no external funding.
**RECORD 22.1**	Explain how supplemental information such as protocol, raw data, or programming code can be accessed.	11, 15-17; supplementary files	The Data Availability Statement explains HCUP licensing and non-redistribution. File S1 provides non-data-bearing SAS analysis programs, Appendix B provides the variable dictionary/code sets, and File S2 provides this completed STROBE/RECORD checklist; materials are also available from the corresponding author.
